# Adherence Scale to the Recommendations for Mental Health during the COVID-19 pandemic from the Portuguese General Directorate of Health (ASR-MH-COVID19) - Development and validation

**DOI:** 10.1192/j.eurpsy.2022.679

**Published:** 2022-09-01

**Authors:** A.T. Pereira, C. Cabacos, S. Soares, M.J. Pacheco, A. Manão, A. Araújo, A.P. Amaral, R. Sousa, A. Macedo

**Affiliations:** 1 Faculty of Medicine of University of Coimbra, Institute Of Psychological Medicine, Coimbra, Portugal; 2 Coimbra Institute for Biomedical Imaging and Translational Research, -, Coimbra, Portugal; 3 Centro Hospitalar e Universitário de Coimbra, Department Of Psychiatry, Coimbra, Portugal; 4 Institute Polytechnic, Coimbra Health School, Coimbra, Portugal, Coimbra, Portugal; 5 Fernão de Magalhães Health Center, Ars Centro, Coimbra, Portugal

**Keywords:** Covid-19, Adherence Scale to the Recommendations for Mental Health during the COVID-19

## Abstract

**Introduction:**

The COVID-19 crisis has generated an increasing stress throughout the population.

**Objectives:**

To develop and validate the **A**dherence **S**cale to the **R**ecommendations for **M**ental **H**ealth during the **COVID-19** pandemic from the Portuguese **G**eneral **D**irectorate of **H**ealth (GDH) (ASR-MH-COVID19).

**Methods:**

The items content was based on the GDH guides for the prevention of mental health and psychosocial well-being of the general population during the COVID-19 outbreak. After content and facial validity analysis, the preliminary version of the ASR-MH-COVID19 (8 items to be answered on a Likert scale) was completed by 413 individuals (69.2% female; mean age=31.02±14,272), in September-December 2020 (Sample1) and then by 967 (70.9% female; mean age=34.02±14,272), in February-May 2021 (Sample2). Sample1 was randomly divided in two sub-samples. Sample1A was used for exploratory factor analysis/EFA and Sample1B for confirmatory factor analysis/CFA; CFA was then replicated with Sample2. The online surveys also included the **A**dherence **S**cale to the **R**ecommendations of Portuguese GDH to minimize the impact of COVID-19 (ASR-COVID-19; Pereira et al. 2020).

**Results:**

CFAs were informed by EFA and showed that the unidimensional model presented acceptable-good fit indexes (Sample1B: χ^2^/df=2.747; RMSEA=.0980, p<.001; CFI=.973; TLI=.918, GFI=.972; Sample2: χ^2^/df=3.327; RMSEA=.0490, p<.001; CFI=.993; TLI=.983, GFI=.990). Cronbach’s alfas were α<.850. Pearson correlations between ASR-MH-COVID19 and ASR-COVID19 were significant (p<.01) and moderate-high for the total (r=.753) and dimensional scores (Distance and respiratory hygiene, r=.739; House and personal hygiene, r=.584; Use of remote services and isolation r=.425).

**Conclusions:**

The new ASR-MH-COVID19 has shown validity and reliability, allowing the investigation of this (mental) health behaviour.

**Disclosure:**

No significant relationships.

